# Supplementary material for: Smartphone Apps Targeting Hazardous Drinking Patterns among University Students Show Differential Subgroup Effects over 20 Weeks: Results from a Randomized, Controlled Trial
Source: J Clin Med. 2019 Oct 28;8(11):1807. doi: 10.3390/jcm8111807 (PMC6912621; doi:10.3390/jcm8111807)
Supplement: Supplementary file 1 [file jcm-08-01807-s001.pdf]

Table S1. Response parameters of Hidden Markov Model.

Hidden state/ drinking level [a]	Abstinent	Moderate	Excessive
Monday	0.95	0.04	0.01
Tuesday	0.91	0.07	0.02
Wednesday	0.75	0.21	0.04
Thursday	0.86	0.11	0.03
Friday	0.29	0.37	0.33
Saturday	0.25	0.33	0.42
Sunday	0.92	0.08	0.01
**Frequent-heavy drinkers**	**0.32**	**0.49**	**0.20**

a. Response parameters were proportions of abstinence, moderate drinking and excessive drinking per hidden state.

Table S2. Transition Matrix for the eight latent classes identified in the Hidden Markov analysis of 2166 university students with moderate or excessive alcohol use, participating in randomized controlled app trials to reduce consumption.

To: → From: ↓	Monday	Tuesday	Wednesday	Thursday	Friday	Saturday	Sunday	Frequent-heavy drinkers
Sunday	1							
Monday		1						
Tuesday			1					
Wednesday				1				
Thursday					0.99	0.01		
Friday						1		
Saturday							1	
**Frequent-heavy drinkers**					**0.01**			**0.99**

Table S3. Associations between Promillekoll intervention group and drinking days within the subgroup of frequent-heavy drinkers (n=146).

	**Drinking day (Y/N)**			
	Odds Ratio	*CI*	*std. Error*	*p*
**Fixed Parts**				
(Intercept)	2.28	1.70–3.07	0.34	<0.001
Control*	0.66	0.46–0.96	0.12	0.029
PartyPlanner*	0.90	0.58–1.41	0.20	0.650
Time	0.98	0.97–1.00	0.01	0.052
Age	1.01	1.00–1.02	0.01	0.029
Sex	0.98	0.79–1.21	0.10	0.833
Control* X Time	1.03	1.01–1.05	0.01	0.004
PartyPlanner* X Time	1.01	0.99–1.04	0.01	0.270
**Random Parts**				
$\tau_{00, id}$	0.179			
N_{id}	146			
ICC_{id}	0.052			
Observations	3381			
Deviance	4161.433			

Note. * Promillekoll is the reference category. The fitted model is a generalized linear mixed model with a binomial link function (R library lme4). A random intercept is fitted for each participant in the analysis. A random slope model did not lead to a better fit based on anova comparison, and overall led to very similar results. The dependent variable Drinking day indicates whether the participant drank alcohol on each of the 28 days for which data were collected. See also Figure 4a.

Table S4. Associations between TeleCoach group and drinking days within the subgroup of frequent-heavy drinkers (n=146).

	**Drinking day (Y/N)**			
	Odds Ratio	***CI***	***std. Error***	***p***
**Fixed Parts**				
(Intercept)	3.12	1.80–5.39	0.87	<0.001
Assessment-only*	0.54	0.31–0.96	0.16	0.037
Wait list*	0.79	0.36–1.76	0.32	0.568
Time	0.95	0.93–0.98	0.01	0.001
Age	1.01	1.00–1.02	0.01	0.058
Sex	0.99	0.80–1.22	0.11	0.916
Assessment-only* X Time	1.06	1.03–1.09	0.02	<0.001
Wait list* X Time	1.03	0.99–1.08	0.02	0.144
**Random Parts**				
$\tau_{00, id}$	0.186			
N_{id}	146			
ICC_{id}	0.054			
Observations	3381			
Deviance	4150.276			

Note. * TeleCoach is the reference category. The fitted model is a generalized linear mixed model with a binomial link function (R library lme4). A random intercept is fitted for each participant in the analysis. A random slope model did not lead to a better fit based on anova comparison, and overall led to very similar results. The dependent variable Drinking day indicates whether the participant drank alcohol on each of the 28 days for which data were collected. See also Figure 4b.
